# Shared resources, shared costs—leveraging biocuration resources

**DOI:** 10.1093/database/bav009

**Published:** 2015-03-16

**Authors:** Sandra Orchard, Henning Hermjakob

**Affiliations:** European Molecular Biology Laboratory, European Bioinformatics Institute (EMBL-EBI), Wellcome Trust Genome Campus, Hinxton, Cambridge CB10 1SD, UK

## Abstract

The manual curation of the information in biomedical resources is an expensive task. This article argues the value of this approach in comparison with other apparently less costly options, such as automated annotation or text-mining, then discusses ways in which databases can make cost savings by sharing infrastructure and tool development. Sharing curation effort is a model already being adopted by several data resources. Approaches taken by two of these, the Gene Ontology annotation effort and the IntAct molecular interaction database, are reviewed in more detail. These models help to ensure long-term persistence of curated data and minimizes redundant development of resources by multiple disparate groups.

**Database URL:** http://www.ebi.ac.uk/intact and http://www.ebi.ac.uk/GOA/

## Introduction

The volume of data being generated by biological laboratories world-wide is becoming ever greater, with high throughput technologies, instrumentation and data analysis techniques increasing in efficiency every year. The end point data from large-scale, publicly funded studies such as ENCODE (1), 1000 Genomes (2) and the Human Proteome projects (3) are available for research groups world-wide to download, re-analyze and to use to formulate and test novel hypotheses. As data quantity increases however, the mechanisms put in place to store, annotate and process this information become more critical and need to become more sophisticated. Accurate recording of experimental data is critical with not only the results being captured in an appropriate format, but also the corresponding experimental conditions. The measurement of changes in transcript or protein level, for example, is of limited use if the meta-data describing the potential reasons for the observed changed are not also detailed and stored alongside. There have been many publications describing the importance of these procedures, and proposing the use of ontologies to consistently annotate data from disparate sources. Making data available in community agreed standardized formats to enable data portability and facilitate integration with information from related studies has also been argued for many times, and most databases are now built based on these fundamental concepts. However, the addition of meta-data to any dataset is time-consuming and requires the input of a trained biocurator. Despite this obvious need, such posts are often difficult to fund in times when research budgets are under pressure. In addition, specialist editorial tools and data maintenance procedures are required, to firstly add the value-added information to the original dataset and to subsequently maintain both dataset and meta-data in line with changes to our understanding of underlying reference resources such as genome/protein sequences or updates to the ontologies used to annotate the information. Again these tools and procedures are expensive to establish and maintain and it is often difficult to obtain funding to even produce them in the first place.

## Is manual curation justified?

Employing a skilled biocurator to add value to a dataset is undeniably expensive and there are many who would argue that the same processes can be automated, could be handled by the submitting author or the same data gathered in using text-mining procedures. However, there are many examples in the field of biomedical databases in which manual curation and automated annotation work in parallel and serve to prove the value of the trained biocurator. The UniProtKB/Swiss-Prot database has been cited as the gold standard of information content for many years and relies on biologists reading papers, identifying and extracting the key information and adding this to the appropriate protein sequence entry in a structured format (4). At the time of writing, UniProtKB contained over 90 million entries and is growing exponentially. Most of the proteins represented in the database have only been predicted to exist based on gene models from sequenced DNA or RNA and have not been experimentally verified at the protein level. Computational procedures are used to group closely related, well annotated proteins in UniProtKB/Swiss-Prot, identify the annotation common to all of them and transfer this to unreviewed proteins in UniProtKB/TrEMBL (5). A simple comparison of an entry in UniProtKB/Swiss-Prot, for example the entry for human Beta-hexosaminidase (P07686) with the orthologous entry for the closely related chimpanzee which has not yet been manually annotated (H2QR30), allows the researcher to very easily see the value added by the biocurator. Automated annotation is a valuable tool, which adds basic information to entries for which there is no experimental evidence, but the entries lack the detail which is selected as relevant to that species and manually added to the entry. Similar observations can be made when looking at the granularity of Gene Ontology (GO) annotation added computationally to gene products by automated annotation. The K-ras protein in mouse (P32883) has detailed manual annotation attached to it such as ‘positive regulation of Rac protein signal transduction (GO:0035022) and “actin cytoskeleton organization (GO:0030036)” whereas the equivalent entry in rat (A0JN17), currently only displays the predictive annotation “GTP binding (GO:0005525)” and GTP catabolic process (GO:0006184)’. Whilst automated annotation is an essential tool to give the researcher indications of the role a particular protein plays in a poorly studied organism, it cannot compare to the level of information added by manual curation achieved by a detailed study of the relevant literature. It should also be noted that since automated annotation is largely a process of information transfer, there can be no automated annotation without detailed curation first being manually added to well-studied sequences. This makes it even more critical that stringent quality control procedures are in place to ensure the veracity of the original information capture, since errors will not only affect the original entry but potentially also be proliferated across multiple closely related records.

Entry annotation by the submitting author is an attractive argument for those databases where data deposition is the primary information gathering mechanism and has been attempted by several communities. The MINT database reported on an experiment to encourage authors to write ‘structured digital abstracts’ (SDAs) when submitting interaction data to FEBS Letters (6). Authors voluntarily filled in a spreadsheet to report their protein interaction data in minimalistic terms using protein identifiers and controlled vocabulary (CV) terms. A series of paragraphs were then generated, each of which contained a relationship between two biological entities qualified by the experimental method that was used to support the relationship. Authors, however, proved unenthusiastic about cooperating even with these relatively simple requirements, and the SDAs are now generated by the MINT curators themselves ahead of publication of the paper. Other communities have, however, proved more cooperative and PomBase, the fission yeast database, have developed a web-based tool to provide a curation interface for both curators and researchers, to support community curation (7). This approach in general appears to be more successful when the researcher community is relatively small, and therefore easily accessible on a personal level by the database, although entries often still need input from a trained curator before going public.

It has often been stated that text-mining is an acceptable substitute for manual curation. The BioCreative: Critical Assessment of Information Extraction in Biology challenge was established in 2003 as an international community-wide effort for evaluating text-mining and information extraction systems applied to the biological domain (http://www.biocreative.org/). This group has now run a series of data gathering challenges based on specific text-mining exercises (8), which were reviewed and evaluated at four public workshops. Several tools have been designed specifically for the competition, others have been refined to meet BioCreative tasks, however despite all this effort, there has not been a notable replacement of manual curation by text-mining. In cases where text-mining tools are being used, for example Textpresso which has been adopted by several model organism databases (9), these are largely only used for triaging papers and highlighting those appropriate for subsequent manual curation. The precision and accuracy achieved by these tools still falls well short of that of a biocurator, and while a significant proportion of data cannot be captured from the original published paper by manual curation because of missing data not supplied by the author, it is difficult to see how text-mining can ever be successful in providing highly detailed annotation.

The real value of manual curation can perhaps be best judged by looking at targeted curation projects. The Gene Ontology Annotation project supplies several examples of this, with the improvement of cardiovascular-focused GO annotations having been demonstrated to have led to an evident improvement of microarray interpretation (10). Analysis of datasets derived from peripheral blood mononuclear cells from patients with systemic scleroderma-related pulmonary arterial hypertension and from mouse macrophages both showed significantly improved enrichment of terms appropriate to both cardiovascular function and disease process. Similarly, the creation of ∼9600 kidney-related GO term associations to 940 UniProtKB entries resulted in significant improvements to the interpretation of analyses performed on genes differentially expressed in kidney glomeruli affected by diabetic nephropathy (11). In a separate effort, the Michael J. Fox Foundation for Parkinson's Research funded the manual annotation of a molecular interaction network centred on LRRK2, a leucine-rich protein kinase, mutations in which are a frequent cause of autosomal dominant familial Parkinson’s disease (12). Analysis of the resulting interaction network has similarly led to increased understanding of the disease and the pathways and processes associated with it and this significantly improved network is now available to enable analysis of ‘Omics’ datasets generated from populations suffering from this condition and will enable novel testable hypotheses to be generated to further our understanding of this disease.

## Sharing curation effort

As costs rise, and grant money becomes increasingly difficult to obtain, it is becoming more and more important that data resources examine their practices and identify areas in which curation efforts overlap with, or are redundant to, parallel activities undertaken by other resources. ‘Competitors’ are more productively regarded as potential ‘collaborators’ and it is a better service to both the tax payer, who ultimately funds database activities, and the researcher if data resources maximize the return of investment by sharing curation efforts rather than redundantly repeating the same activities in isolation. It is also more efficient if areas of specialist interest, for example protein structures, molecular interactions and mass spectrometry, are handled by data providers who understand these data, who can process and filter these data types and provide appropriate exports/cross references to more generalist resources, rather than for these databases attempt to handle these data types themselves. There is a long history of data sharing in the biomedical community—the Protein Data Bank (13) and International Nucleotide Sequence Database Collaboration (14) have both existed for over 35 years and provide multiple data deposition/search points for single united datasets of three-dimensional structural data of large biological molecules and the results of nucleic acid sequencing efforts respectively. ProteomeXchange is a more recent addition to this group of archival databases, and provides a coordinated submission of MS proteomics data to the main existing proteomics repositories (15). In all these cases, the data are subsequently made available in a community accepted data formats and can be downloaded by the researcher for reanalysis and evaluation using a range of tools which have been specifically developed to read these formats.

More recently, secondary databases which require a higher degree of manual intervention than do the archival databases have also been uniting to share curation load. The GO project was one of the front runners in establishing a collaborative effort to address the need for consistent descriptions of gene products across databases. Founded in 1998, the project began as a collaboration between three model organism databases, FlyBase (Drosophila), the Saccharomyces Genome Database (SGD) and the Mouse Genome Database (MGD) but now encompasses the efforts of over 30 manually curated resources world-wide (16, 17). GO annotations added by curators at all of these databases, using rules agreed to by the consortium members, are centrally collated into the GO database. In the database, the data are actively maintained with, for example, annotations made to obsoleted terms being removed and remapped, then the merged dataset is made available to resources such as UniProtKB and the genome browsers in an agreed file format. Similarly, the International Molecular Exchange (IMEx) consortium has more recently started to share the curation of protein– protein interactions. Where previously a paper could be redundantly curated in multiple databases, the consortium now manage their efforts centrally and ensure that a publication is curated only once (18). Again the consortium have agreed the rules by which the curation is undertaken, and community formats exist in which the data are made publicly available. The IMEx dataset is therefore a non-redundant set of consistently curated records made available in an established data format, and the records are also made available for search and download as a distinct dataset through a web service protocol, PSICQUIC (19). This non-redundant set of records, or specific subsets of them, are available for network analysis or for representing in other resources, for example Mentha (20) and VirusMentha (21). One important aspect of the IMEx Consortium is that it has included a mechanism for ensuring that loss of a member database due to funding cuts or a PI moving on does not inevitably lead to the loss of the data within it. All IMEx partners agree that their data must be made available for import by another partner resource if they leave the consortium for any reason. This mechanism successfully ensured that data within the Microbial Protein Interaction Database (22) is still actively maintained and available via the IntAct database, several years after the database has ceased to be actively maintained at the J. Craig Venter Institute.

**Figure 1.**
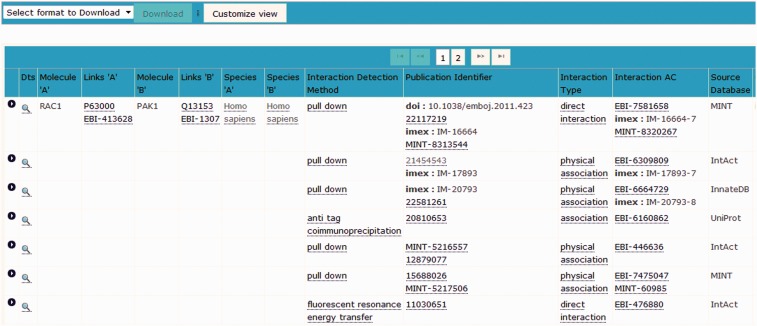
The institute manager within the IntAct database enables full accreditation of the effort of contributing curation groups to be displayed within each record.

## Shared tool development, shared infrastructure

Establishing the infrastructure required to run a production database and maintain update cycles in line with the appropriate underlying reference databases is an expensive procedure, as is building and maintaining a dedicated editorial tool. Such a commitment is beyond many groups and numerous databases fail to survive past the life-cycle of their initial funding, often leading to a loss of the data captured within them. A small resource which may only consist of a single developer who is responsible for the database environment and website production may be unable to undertake any additional tool development. One model that has been successfully followed by several groups is that of a single central database being created to hold and maintain the data with a web-based editorial tool enabling many groups to add to the dataset it contains.

**Figure 2.**
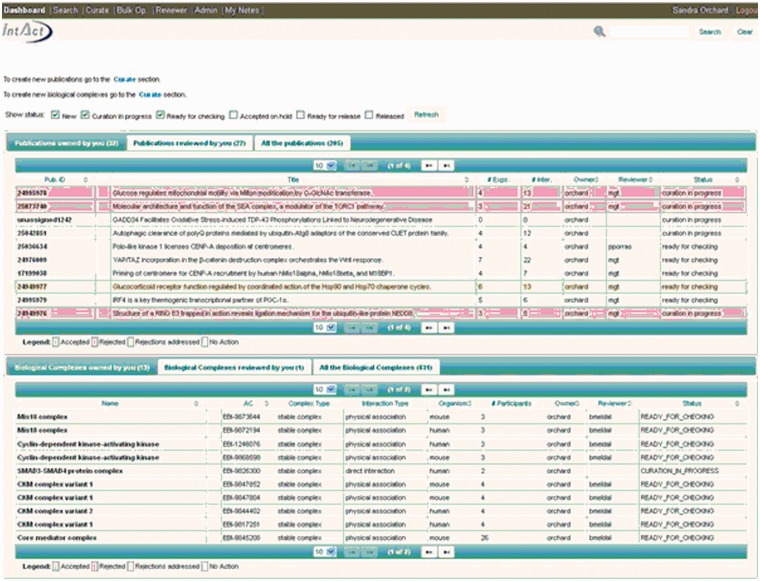
The IntAct web-based editorial tool.

The IntAct molecular interaction database provides such a curation platform for several other data resources interested in capturing experimentally derived interaction data, mainly, but not exclusively protein–protein interaction evidences. Many of these groups are very small, and interested in capturing data from a specific area of biology in which they have particular expertise but lack the funding and infrastructure to establish an independent data resource and curation environment. The IntAct curation system is a web-based platform, designed to allow collaborative curation by physically remote partners, including UniProtKB/Swiss-Prot curators based in Geneva, Switzerland, the MINT database curators and also the MatrixDB curation team (23). IMEx-level curation requires the mapping of binding regions, point mutations and post-translational modifications to a specified sequence within a reference protein sequence database, UniProtKB in the case of IntAct. An update to a predictive gene model may result in a corresponding change to the protein sequence(s) derived from it. Interactions involving domains and/or residues of that protein sequence then require a corresponding update to ensure that the mapping to the updated sequence is correct. Update pipelines need to be run regularly, in line with the release cycle of the sequence database, namely every 4 weeks in the case of UniProtKB. Similarly, CVs used to annotate interaction data need to be refreshed with every new release (24). This is a computationally complex set of procedures run by the IntAct team at the European Bioinformatics Institute on the entire dataset within IntAct. The editor contains an institute manager module, which means that each individual curator can be associated with a specific institute or granting body, enabling full attribution of the data to the funding source (Figure 1). As a consequence of this, each contributing group can also be provided with its own PSICQUIC web service (25) running from within the IntAct database, which can be embedded within a web page or tool, completely independently of other data present in the database. Alternatively groups may choose to regularly download selected files from the IntAct ftp site for import into their own resource. Each curation team can therefore have access to a sophisticated editorial tool (Figure 2) and have their data updated and maintained, and easily accessible to them, without having to invest in the infrastructure to provide this. The detailed information captured in the combined dataset allows for sophisticated filtering of the data to ensure that only the highest confidence interaction sets are exported back out to third party resources such as UniProtKB, GO annotations and neXtProt (26). The IntAct team have recently extended the editorial interface to enable the curation of reference sets of protein complexes. This involves different, but overlapping, curation teams who are collaborating to provide a single centrally maintained resource, the Complex Portal, which makes protein complexes available to be imported into, or cross-referenced from, other data resources (27).

Similarly, the UniProt-GOA’s annotation tool, Protein2GO (28), is already used by several additional groups such as the UCL Functional Gene Annotation group as a common biocuration platform and this resource is being further developed, both to increase its accessibility to additional annotation groups and also to enable annotation to additional molecule types, with the protein complexes described above being the first example of this. Again, this is enabling relatively small communities with expertise in the genome of a particular organism, or family of organisms, to contribute to the global curation effort without a major infrastructure investment. The InterMine group have taken this a stage further and designed a database platform independently of any model organism or community annotation group but which is appropriate to be used by any of these. The generic data warehouse has been built specifically for the integration and analysis of complex biological data and enables the creation of biological databases accessed by web query tools (29). The web interface is designed to be easily customized; a scriptable web-service allows programmatic access to the data. The InterMine database build system allows for the integration of datasets with modules that load data from common biological formats (e.g. GFF3, FASTA, OBO, BioPAX, GAF, PSI-MI) with an identifier resolver system that can map different identifiers, including outdated accessions, to a single reference set. This data warehouse has been adopted by many model organisms as a means to handle large datasets, with the InterMine development team funded to provide updates to the software as data types evolve. Again, this is an example of a single centralized development effort then being leveraged by multiple groups, at minimal cost to themselves.

## Summary

Although manual curation of biological data is an expensive process, it is without question a necessary procedure for almost every biomedical database and there are many examples where the value added by such activities are demonstrable and even measurable. It is in the interest of the databases wishing to employ biocuration procedures to ensure best value for the investment made by funding bodies by ensuring that they work with existing resources available in the community, rather than ‘reinventing the wheel’ by redundantly developing multiple curation tools to annotate the same datasets. New databases are being established all the time and, whilst this should be seen as a positive move bringing novel ideas and new directions into established fields, it is regrettable that many of these fail to survive beyond the initial grant funding period. Funding bodies should be encouraged to favour grant applications which both signal an intention to work with existing communities to improve and adapt their tools for novel uses, and also those which have a data maintenance plan, to ensure the data remain both current and in the public domain should the database for any reason cease operation. The International Society for Biocuration (www.biocurator.org/*)* provides a forum, through its annual conference, for web-based tools to be described and demonstrated to potential adopters and perhaps could play a wider role in advertising the availability of these resources throughout the year. In times of economic hardship, it is in the interests of all curation resources to make optimal use of data and tool sharing efforts and for larger, comparatively well-funded resources to support the activities of smaller communities who can add valuable, domain-specific information to a larger common dataset.

## Funding

This work was funded by the European Commission under Affinomics [FP7-241481], BBSRC MIDAS grant (BB/L024179/1) and NHLBI Proteomics Center Award [HHSN268201000035C]. Funding for open access charge: European Molecular Biology Laboratory (EMBL).

*Conflict of interest*. None declared.
